# The phenotypic variability of *HK1*-associated retinal dystrophy

**DOI:** 10.1038/s41598-017-07629-3

**Published:** 2017-08-01

**Authors:** Zhisheng Yuan, Baiyu Li, Mingchu Xu, Emmanuel Y. Chang, Huajin Li, Lizhu Yang, Shijing Wu, Zachry T. Soens, Yumei Li, Lee-Jun C. Wong, Richard A. Lewis, Ruifang Sui, Rui Chen

**Affiliations:** 1 Department of Ophthalmology, Peking Union Medical College Hospital, Peking Union Medical college, Chinese Academy of Medical Sciences, Beijing, China; 2 MOE Key Laboratory of Contemporary Anthropology, School of Life Sciences, Fudan University, Shanghai, China; 3 Department of Molecular and Human Genetics, Baylor College of Medicine, Houston, TX USA; 4 Human Genome Sequencing Center, Baylor College of Medicine, Houston, TX USA; 5 Retina and Vitreous of Texas, Houston, TX USA; 6 Department of Ophthalmology, Cullen Eye Institute, Baylor College of Medicine, Houston, TX USA

## Abstract

Inherited retinal dystrophies (IRDs) are a clinically and genetically heterogeneous group of Mendelian disorders primarily affecting photoreceptor cells. The same IRD-causing variant may lead to different retinal symptoms, demonstrating pleiotropic phenotype traits influenced by both underlying genetic and environmental factors. In the present study, we identified four unrelated IRD families with the *HK1* p.E851K variant, which was previously reported to cause autosomal dominant retinitis pigmentosa (RP), and described their detailed clinical phenotypes. Interestingly, we found that in addition to RP, this particular variant can also cause dominant macular dystrophy and cone-rod dystrophy, which primarily affect cone photoreceptors instead of rods. Our results identified pleiotropic effects for an IRD-causing variant and provide more insights into the involvement of a hexokinase in retinal pathogenesis.

## Introduction

Inherited retinal dystrophies (IRDs) are a group of Mendelian diseases caused by photoreceptor dysfunction and cell death. IRDs demonstrate both extensive clinical and genetic heterogeneity. To date, nearly 300 genes have been identified that associate with various forms of IRDs (https://sph.uth.edu/retnet/) (accessed Jun 23^rd^, 2017) and they are involved in a wide range of biological pathways such as photo-transduction^1, 2^, ciliogenesis^3, 4^, pre-mRNA splicing^5, 6^, transcription regulation^7^ and protein transport/quality control^8, 9^. Nevertheless, novel disease-causing genes and pathways are still being identified by whole exome sequencing or whole genome sequencing, adding to the pathological mechanisms of retinal dystrophy.

Different types of IRDs can be caused by mutations in the same gene. For example, mutations in *ABCA4* can lead to Stargardt disease, cone-rod dystrophy (CRD), or retinitis pigmentosa (RP), which differentially affects cone and/or rod photoreceptors^10–12^. Furthermore, one specific disease-causing variant may also lead to different clinical presentations of IRD. The *PRPH2* p.P210R mutation, which was identified in autosomal dominant disease families, displays a broad phenotypic variability ranging from macular to peripheral retinal dystrophy^13, 14^. This phenomenon demonstrated the complexity of IRD genotype-phenotype correlations and that modifier alleles or environmental factors may play important roles in determining the phenotype of a patient.

One missense mutation affecting the human hexokinase 1 (*HK1*, NM_033497.2, c.2551 G > A, p.E851K) was previously identified in multiple families to associate with autosomal dominant retinitis pigmentosa^15, 16^. Here, by targeted capture sequencing of known IRD-associated genes, we report four additional IRD cases with this variant, further confirming the disease association and supplementing the described phenotypic variability of *HK1*-related retinal dystrophy.

## Results

### Genetic findings

**Figure 1:**
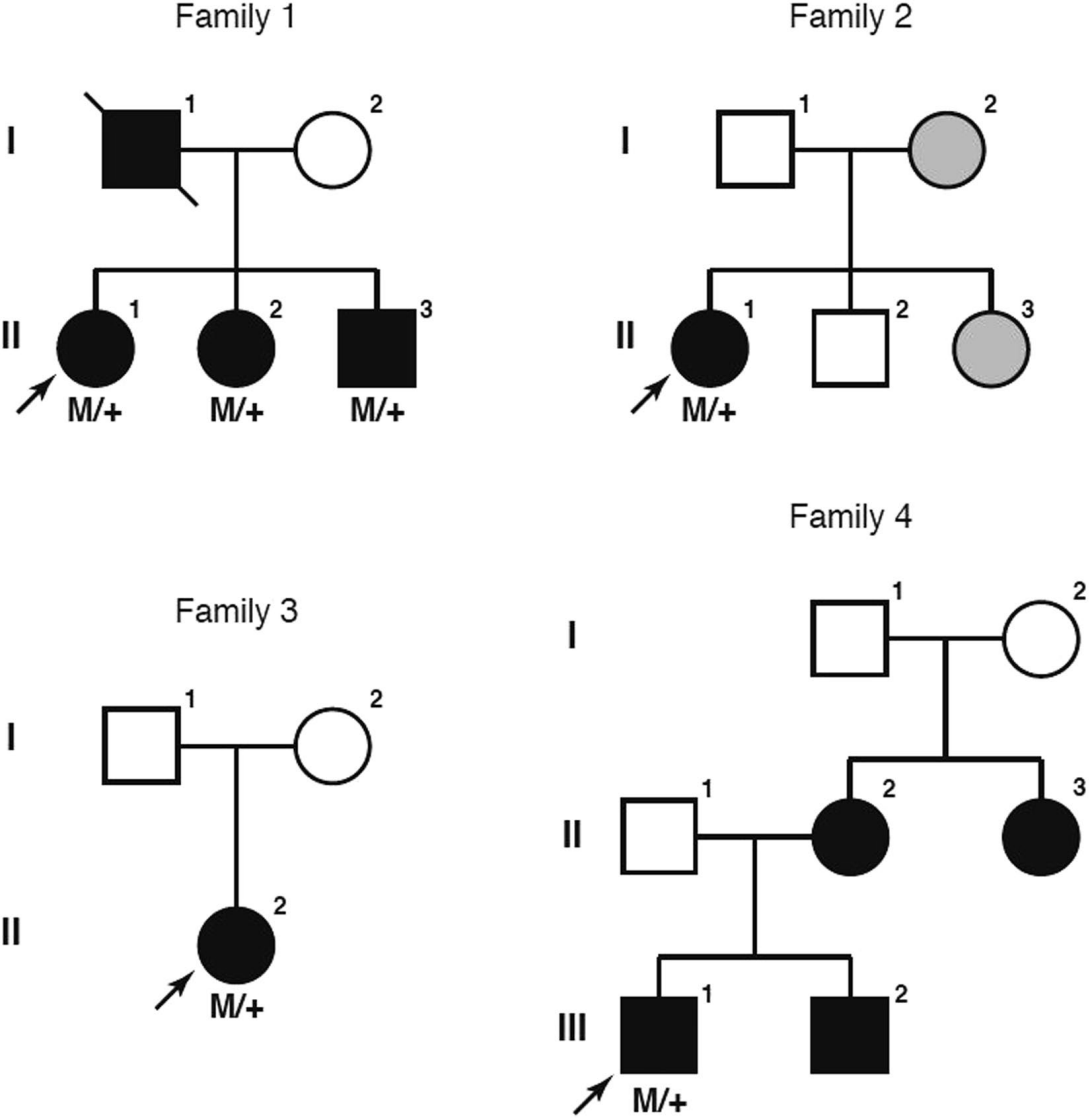
The pedigrees of the four IRD families in this study. “M” indicates the *HK1* p.E851K mutation. Arrow indicates the proband. Grey coloring in the Family 2 indicates an unknown retinal phenotype.

A total of four unrelated IRD probands (one Asian and three Caucasians) were found to possess the previously reported *HK1* p.E851K variant by target capture panel sequencing. High quality next-generation sequencing data were obtained for each sample (mean coverage: 128.7; coverage ≥ 10×: 97.5%) and no other plausible disease-causing variants were identified. Additional rare protein-coding variants identified are summarized in Supplementary Table 1. Sanger sequencing was performed to confirm this variant and genotype-phenotype co-segregation. The pedigrees of these four families are shown in Fig. 1.

We then explored the disease-associated haplotype of these four families and the previous published *HK1* disease family^16^. As shown in Supplementary Table 2, the Han Chinese Family 1 in this study possesses a haplotype clearly different from the previously reported Caucasian family outside the 16 kb window between rs749105 and rs14006. This result strongly suggested that the *HK1* E851K variant is *bona fide* the disease-causing variant.

### Clinical findings

**Figure 2:**
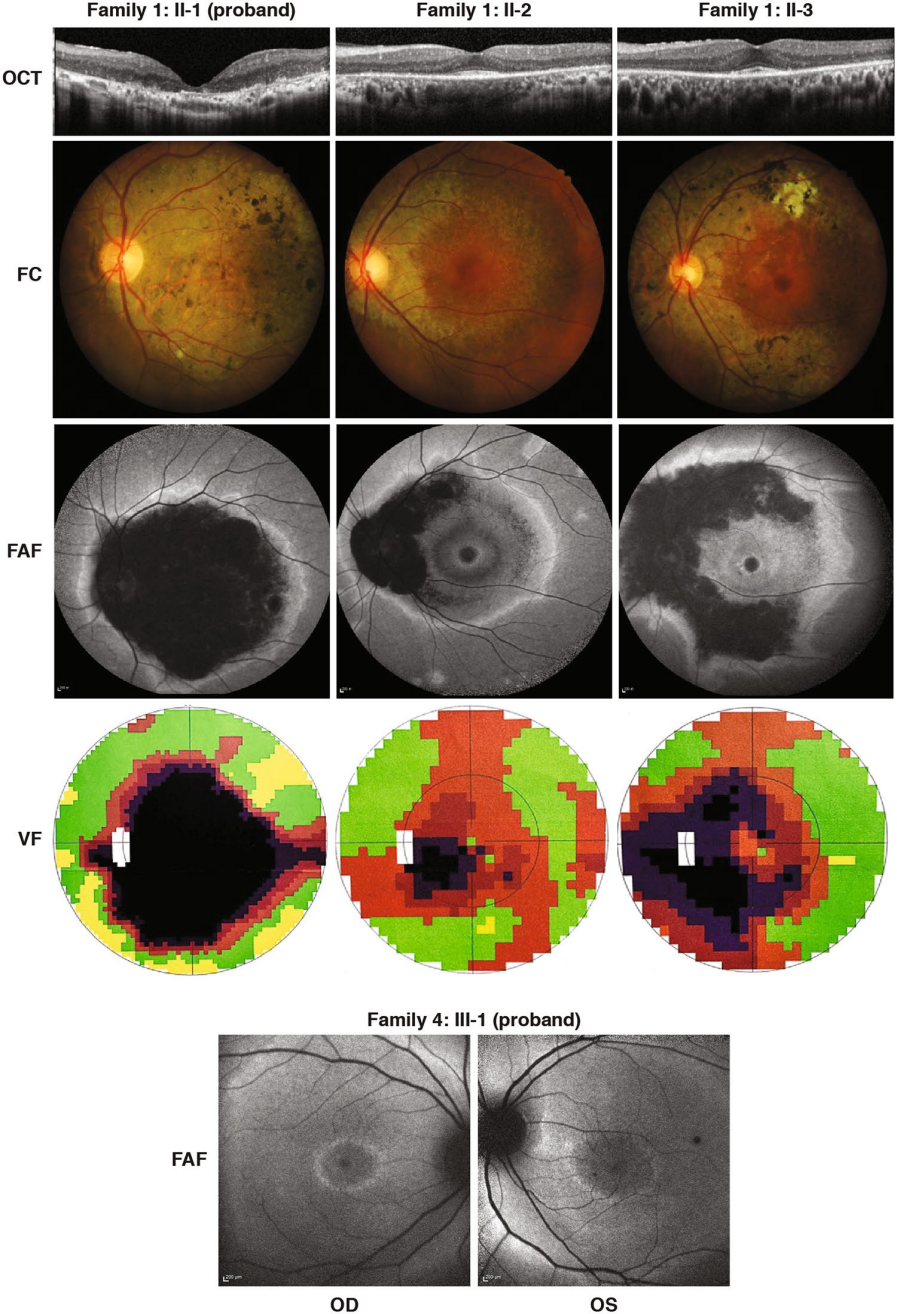
The clinical phenotypes of the patients. The phenotypes of the three affected individuals in Family 1 and the proband in Family 4. OCT, optical coherence tomography; FC, ophthalmoscopy; FAF, fundus autofluorescence; VF, visual field. OD, the right eye; OS, the left eye. Note all the pictures for Family 1 show the phentoypes of the left eye.

The clinical features of all the patients are summarized in Table 1. Family 1 (F1) is of the Han Chinese ethnicity. There are four patients in this family presenting a dominant inheritance pattern. The proband F1:II-1 is a 54 year-old female. Since the initial diagnosis of macular dystrophy at the age of 24, she has been followed up for 30 years. Her central visual acuity has progressively worsened to its current state of hand motion OU (*oculus uterque*, both eyes). Recently, detailed ophthalmologic examinations were performed in all three available affected family members and representative clinical phenotypes are shown in Fig. 2. The proband’s color vision is impaired and her fundus images showed features typical of macular dystrophy features including posterior pole atrophy with scattered pigment deposits throughout the retina of both eyes. In accordance with the ophthalmoscopy result, optic coherence tomography (OCT) revealed the disappearance of the ellipsoid and interdigitation zones in both eyes. Bilateral large central scotomas were present in her 30 degree visual field exams. Electroretinogram (ERG) results showed rod (scotopic 0.01) and rod-cone combined (scotopic 3.0) responses were normal, and cone (photopic 3.0 and 30 Hz flicker) responses were mildly reduced in both eyes. However, different from the macular dystrophy phenotype in the proband, the patient F1:II-2 was diagnosed as CRD and F1:II-3 was diagnosed as RP. F1:II-2 is a 50 year-old female who has complained about poor darkness adaptation for 15 years. The diagnosis of CRD was confirmed by the ERG results with mildly reduced rod and rod-cone combined responses, as well as moderately reduced cone responses. The best corrected vision acuity (BCVA) of patient F1:II-2 is 20/25 for OD (*oculus dexter*, the right eye) and 20/30 for OS (*oculus sinister*, the left eye). Patient F1:II-3 is a 48-year-old male. Since his initial onset of RP, he has suffered from night blindness for about 20 years and reduced visual acuity for 4 years. His best corrected vision acuity is 20/30 (OU). Apparent retinal atrophy and bone-spicule pigmentary deposits along and beyond the arcade with a normal fovea were observed in his ophthalmoscopy. OCT images revealed the loss of the ellipsoid and interdigitation zone except for in the foveal area, with increased foveal thickness in both eyes. His visual field test demonstrated a constricted visual field. The ERG results showed bilateral remarkably reduced rod and rod-cone combined responses, and moderately reduced cone responses. The anterior segments of all three patients was normal. As for the deceased patient F1:I-1, an early-onset macular dystrophy was reported.

**Table 1:** Ophthalmological findings in the patients.

Patient	Eth	Age	AgeDx	BCVA	ERG	VF	OCT	OphCP and FA
F1: II-1	HCh	54	18	HM OU	Rod: N Cone:+	central scotoma OU	Absence of EI zones OU	posterior pole atrophy OU; PEA OU;
F1: II-2	HCh	50	35	20/25 OD 20/30 OS	Rod:+ Cone:++	arcuate visual defect OD; para-central scotoma OS	Absence of EI zones OU except fovea	ring shaped atrophic posterior pole with normal fovea OU
F1: II-3	HCh	48	28	20/30 OU	Rod:+++ Cone:++	constricted VF OU	Absence of EI zones OU except fovea	bone-spicule pigmentary deposits with normal fovea OU; VA
F2: II-1	Cau	38	18	20/30 OU	Rod:++ Cone:++	constricted VF OU	NA	Bone spicules OU; mild VA; mild diffuse ONP OU; PEA
F3: II-1	Cau	37	15–20	20/30 OD 20/25 OS	NA	NA	NA	Bone spicules OU; apparent VA; mild diffuse ONP OU
F4: II-1	Cau	14	10	NA	NA	NA	Bilateral retinal thinning	Mild CME OS; mild VA; optic nerve pallor OU

Eth, ethnicity; HCh, Han Chinese; Cau, Caucasian; AgeDx, Age of diagnosis; BCVA, best corrected visual acuity; HM, hand motion; OU, both eyes; OD, the right eye; OS, the left eye; NA, not available; ERG, electroretinogram; +, mild reduction;++, moderate reduction;+++, severe reduction; VF, visual field; OCT, optical coherence tomography; EI, ellipsoid and interdigitation; OphCP, ophthalmoscopy; FA, fundus autofluorescence; PEA, pigmented epithelial atrophy; VA, vascular attenuation; ONP, optic nerve pallor; CME, cystoid macular edema.

The proband in Family 2 is a 38 year-old Caucasian female. She was diagnosed with RP at the age of 18. Her 63-year-old mother and 34-year-old sister were reported to have “spots” in their retina but the disease phenotype was not confirmed. By age 29, ERG results revealed less than half of normal responses under all testing conditions. At the age of 30, she was aware of progressive constriction of her visual fields and an increasing nyctalopia. Her BCVA was 20/25 OU (OD: −2.50 + 2.00 × 10; OS: −1.75 + 1.25 × 175). Her ocular fundus showed typical features of RP including a mild diffuse optic pallor, mild vascular attenuation throughout, bone-spicule migration and pigmented epithelial atrophy in four quadrants OU. Formal Goldmann visual field tests showed a modest concentric constriction, even with the V4e targets, and large predominantly nasal dense scotomata in each eye. At 34 years, visual field tests demonstrated that a dense circumferential scotoma (III4e) essentially completely surrounds her central 5 degree island in each eye. Her central acuity decreased to 20/30 OU.

The proband in Family 3 is a 37-year-old Caucasian female diagnosed with RP in her late teenage years. She has no known family history of RP. Biomicroscopy showed no abnormality in the anterior segment but notable vitreous syneresis with pigment and cells in each eye, more anteriorly than posteriorly. Her BCVA was stable at 20/30 OD, 20/25 OS, with a mild myopic refractive error: OD, −2.75 + 1.75 × 180; OS, −2.25 + 1.00 × 175. Ophthalmoscopy revealed an atrophic, degenerating retina with apparent vascular attenuation, a mild diffuse pallor of each optic nerve and scattered bone spicules OU.

Family 4 is a dominant RP family of Caucasian ethnicity and the proband is a 14 year-old boy. In the proband, biomicroscopy showed early RP features in the retina and a mild cystoid macular edema in the left eye. Fundus photos revealed very mild vessel attenuation but pallor in the optic nerve of both eyes. Bilateral thinning of the retina and a significant vitreomacular adhesion in the left eye with a cystoid macular edema were striking in his OCT and autofluorescence images. Autofluorescence also showed a hyperfluorescent stressed ring in both eyes (Figure 2). All these observations were in consistent with the diagnosis of RP.

## Discussion

*HK1* encodes hexokinase 1, an important enzyme in the glycolysis pathway^17^. Recessive *HK1* mutations are known to lead to nonspherocytic hemolytic anemia, in which cases hexokinase deficiency is the primary cause^18, 19^ and the phenotype is completely different from IRD. Biochemical studies have shown that the *HK1* p.E851K variant does not affect the hexokinase activity or stability of the HK1 protein^16^. There are also >20 individuals with heterozygous *HK1* protein-truncating alleles in the ExAC database, suggesting that *HK1*-associated dominant IRD probably results from either a gain-of-function or dominant-negative mechanism instead of haploinsufficiency. The disease-causing variant p.E851K is extremely rare but still observed once in the ExAC database (minor allele frequency 1/121,278), which only includes presumably “healthy” controls. This is consistent with our observation of incomplete penetrance in Family 3 and in previous studies^15, 16^. Incomplete penetrance is frequently observed in dominant IRD cases^20–22^. With the availability of genomic variant databases with significantly larger sample size^23^, we are very likely to encounter dominant IRD-causing variants in these control databases at a very low frequency, like *PRPF31* loss-of-function variants and the *HK1* variant in the present study. The variant occurrence, its associated population group, and the disease prevalence will be important for estimating the penetrance of certain dominant IRD-causing variants, thus leading to a more accurate molecular diagnosis. In addition, incomplete penetrance in IRD strongly suggests the effect of additional genetic modifiers, as exemplified by a previous report identifying *CNOT3* in *PRPF31*-associated dominant RP^24^. It is likely that the incomplete penetrance of *HK1*-assoicated IRD also arises from genetic modifiers, which can be further revealed by the recruitment of additional large *HK1*-associated IRD families.

The retina features a high energy consumption, with the particular need for a highly regulated metabolic homeostasis^25^, probably explaining the involvement of metabolic enzymes in retinopathies. The association between glucose metabolism and retinal function has long been studied, and specifically, light versus darkness is known to lead to different rates of glycolysis in the retina^26, 27^. Interestingly, we observed variable phenotypes caused by the *HK1* p.E851K variant including both macular and peripheral retinal dystrophy, suggesting the universality of HK1 in controlling retinal functions. Since HK1 is involved in glycolysis, a ubiquitous biological process rather than a restricted retinal pathway, it is possible that *HK1* genetic defects can be more widely modified by both genetic and non-genetic factors, thus resulting in variable clinical outcomes. Here, by perusing the list of additional protein-coding variants identified by capture panel sequencing, we did not identify variants clearly associated with macular dystrophy phenotype, suggesting that the genetic variation in other genes or intronic variants may be responsible for the phenotypic variability.

In summary, our study identified additional IRD cases with the *HK1* p.E851K mutation, confirming the disease association of this variant. We also report that this variant can be associated not only RP but also macular dystrophy, thus expanding the *HK1*-related phenotypic spectrum. The incomplete penetrance, phenotypic variability, and the probable existence of genetic modifiers associated with this particular IRD-causing variant would provide additional information and guidance on the molecular diagnosis, disease counseling, prediction, and management in the future.

## Methods

### Patient recruitment

The four families investigated in this study were collected from Peking Union Medical College Hospital, Beijing, China or Baylor College of Medicine, Houston, USA. Detailed medical family histories were recorded. Pedigrees of all the patients are drawn based on interview. Detailed ophthalmologic examinations including best visual acuities, color vision tests (D-15 color plates), slit lamp examination, fundus photography, visual field tests, fundus autofluorescence (AF), optical coherence tomography (OCT, Topcon, Tokyo, Japan) (Carl Zeiss, Germany) and Electroretinograms (ERGs, RetiPort ERG System, Roland Consult, Wiesbaden, Germany) were performed on all the patients. ERGs were performed using corneal “ERGjet” contact lens electrodes. The ERG protocol followed the standards published by the International Society for Clinical Electrophysiology of Vision. Genomic DNA was extracted from peripheral blood leukocytes using a commercial kit (QIAamp DNA Blood Midi Kit, Qiagen, Hilden, Germany) according to the manufacturer’s protocol. Written informed consent was obtained from all participating individuals or their guardians. All experimental methods were approved by the Institutional Review Boards of Peking Union Medical College Hospital and Baylor College of Medicine, and were performed in accordance with relative guidelines and regulations.

### Next generation sequencing and data analysis

Pre-capture Illumina libraries were generated as in previous literature^8, 28, 29^. The targeted DNA was captured, washed and recovered using Agilent Hybridization and Wash Kits (Agilent Technologies). The panel sequencing was performed by capturing DNA with probes targeting the exons of known retinal disease-causing genes^30^. Captured DNA libraries were sequenced on Illumina HiSeq. 2000 (Illumina, Inc.). After sequencing, the reads were aligned to human hg19 genome using BWA version 0.6.1^31^. Base quality recalibration and local realignment was performed by the Genome Analysis Tool Kit version 3.6^32^. Atlas-SNP2 and Atlas-Indel2 were used for variant calling^33^. Variant frequency data were obtained from public and internal control databases including the Exome Aggregation Consortium (ExAC) database^23^, CHARGE consortium^34^, ESP-6500^35^ and 1000 Genome Project^36^. Variants with a minor allele frequency higher than the set threshold (1/200 for recessive; 1/10,000 for dominant) were filtered out. Synonymous and deep intronic (distance > 10 bp from exon-intron junctions) variants were also excluded from further analysis. ANNOVAR^37^ (version 06/17/2015) and dbNSFP^38^ (version 2.9, includes SIFT, PolyPhen-2, LRT, MutationTaster, MutationAssessor, etc.) were used to annotate protein-altering effects. Reported retinal disease-causing variants were detected based on the HGMD professional database (version 08/15/2016).

## Electronic supplementary material

Supplementary Information
