# Supplementary material for: The phenotypic variability of HK1-associated retinal dystrophy
Source: Sci Rep. 2017 Aug 1;7:7051. doi: 10.1038/s41598-017-07629-3 (PMC5539152; doi:10.1038/s41598-017-07629-3)
Supplement: Supplementary file 1 — Supplementary Information [file 41598_2017_7629_MOESM1_ESM.pdf]

SUPPLEMENTARY INFORMATION

The phenotypic variability of *HK1*-associated retinal dystrophy

Zhisheng Yuan, Baiyu Li, Mingchu Xu, Emmanuel Y. Chang, Huajin Li, Lizhu Yang, Shijing Wu, Zachry T. Soens, Yumei Li, Lee-Jun C. Wong, Richard A. Lewis, Ruifang Sui, Rui Chen

Family	Chr	Coordinate	REF	ALT	Geno	Freq	Gene	Transcript	Annotation
1	11	17,517,220	A	C	Het	8/246,238	*USH1C*	NM_153676	c.T2551G, p.S851A
1	6	80,203,352	T	C	Het	1/245,890	*LCA5*	NM_181714	c.A836G, p.Q279R
1	6	64,940,499	C	T	Het	16/178,214	*EYS*	NM_001142800	c.G6410A, p.R2137H
1	3	132,407,949	C	T	Het	1/245,986	*NPHP3*	NM_153240	c.G2852A, p.R951Q
1	4	15,534,833	G	A	Het	15/245,884	*CC2D2A*	NM_001080522	c.G1484A, p.R495H
1	9	103,015,286	A	C	Het	Absent	*INVS*	NM_014425	c.A1332C, p.E444D
2	4	123,664,512	G	A	Het	39/277,160	*BBS12*	NM_152618	c.G1465A, p.A489T
2	5	37,198,881	G	A	Het	6/246,250	*C5orf42*	NM_023073	c.C3595T, p.R1199W
2	7	138,603,171	C	T	Het	208/276,898	*KIAA1549*	NM_020910	c.G1201A, p.G401S
3	14	92,347,724	G	T	Het	54/277,116	*FBLN5*	NM_006329	c.C901A, p.L301M
3	4	15,539,735	G	C	Het	469/248,552	*CC2D2A*	NM_001080522	c.G1978C, p.V660L
3	5	82,815,752	G	T	Het	3/276,860	*VCAN*	NM_004385	c.G1627T, p.V543L
4	8	38,871,509	G	A	Het	593/277,054	*ADAM9*	NM_003816	c.G280A, p.V94I
4	1	94,520,793	A	T	Het	3/276,860	*ABCA4*	NM_000350	c.T2461A, p.W821R
4	6	76,657,070	G	T	Het	Absent	*IMPG1*	NM_001563	c.C2005A, p.H669N

Supplementary Table 1. Rare protein-coding variants identified by NGS in this study (in addition to the *HK1* variant)

Chr, chromosome number; REF, reference allele; ALT, alternative allele; Geno, genotype; Het, heterozygous; Freq, gnomAD overall frequency; Genomic coordinates were based on hg19 assembly. All the variants are identified in the proband of the corresponding family. Variant filtering procedure was described in the main text Methods section. Except for the *HK1* variant, no additional putative causative variants were identified.

Chr10 position	Distance (bp)	REF	ALT	SNP	O Freq	EU Freq	EA Freq	Reported	F1	F2	F3	F4
71,142,420	+16,094	G	A	rs748235	0.7757	0.7879	0.6803	BB	AA	AB	AB	AB
71,142,492	+16,002	C	T	rs140498607	0.0048	0.0066	$\approx 10^{-4}$	AB	AA	AA	AA	AA
71,144,702	+13,812	G	A	rs749105	0.6670	0.6880	0.5650	BB	AA	AB	AB	AB
71,149,024	+9,490	G	A	rs117797901	0.0037	0.0007	0.0115	AA	AB	AA	AA	AA
71,152,091	+6,423	T	C	rs2278745	0.5551	0.5204	0.6098	AA	AB	AB	AB	AA
**71,158,514**	**0**	**G**	**A**	**rs777849213**	**$\approx 10^{-5}$**	**$\approx 10^{-5}$**	**$\approx 10^{-4}$**	**AB**	**AB**	**AB**	**AB**	**AB**
71,160,828	-2,314	C	T	rs14006	0.0259	0.0024	0.0317	AA	BB	AA	AA	AA
71,164,692	-6,178	T	C	rs57900755	0.0263	0.0025	0.0319	AA	BB	AA	AA	AA
71,167,047	-8,533	A	G	rs7097827	0.0267	0.0025	0.0322	AA	BB	AA	AA	AA
71,168,766	-10,252	C	G	rs79030584	0.0145	0.0022	0.0321	AA	BB	AA	AA	AA

Supplementary Table 2. *HK1*-associated haplotype information

Original variant calling format (VCF) files of whole exome sequencing or targeted capture sequencing data were used for identifying the disease-associated haplotypes of the previously reported family and four families in the present study. Distance, the genomic distance from the E851K variant; REF, reference allele; ALT, alternative allele; O Freq, overall frequency; EU Freq, non-Finnish European frequency; EA Freq, East Asian frequency. The frequency of alternative alleles were obtained from gnomAD database. Reported, the previously published *HK1* disease family (cited in the main text). F1, Family 1; F2, Family 2; F3, Family 3; F4, Family 4. AA, homozygous reference; AB, heterozygous; BB, homozygous alternative. The information of E851K variant were labeled in bold blue. The genotypes labeled in red indicate the difference of disease-associated haplotype between the reported Caucasian family and the Han Chinese Family 1. Genomic coordinates were based on hg19 assembly.
